# Mild Encephalopathy With Partial Reversible Splenium Lesion Associated With SARS-CoV-2 Infection

**DOI:** 10.7759/cureus.36421

**Published:** 2023-03-20

**Authors:** Cleo Zarina A Reyes, Atef Kokash, Hussam A Yacoub

**Affiliations:** 1 Neurology, Lehigh Valley Health Network, Allentown, USA

**Keywords:** neurology case report, mild encephalopathy with reversible splenium, sars-cov-2, covid-19, mild encephalopathy/aseptic encephalitis with reversible splenial lesion of the corpus callosum

## Abstract

Viral-associated encephalitis/encephalopathy includes a wide spectrum of syndromes reported often in children. A rare form presents with mild encephalitis/encephalopathy and reversible splenial lesion(s). This report describes a case of this rare presentation associated with severe acute respiratory syndrome coronavirus 2 (SARS-CoV-2) infection in a 68-year-old woman. The patient presented to the hospital with altered mental status. Examination revealed mild encephalopathy with disorientation to date and time. Initial laboratory workup was significant for mild hypernatremia and acute kidney injury, and a polymerase chain reaction (PCR) test for SARS-CoV-2 was positive. MRI of the brain revealed an area of hyperintensity and water restriction in the corpus callosum. The patient was treated with tocilizumab, dexamethasone, and remdesivir. MRI of the brain five weeks later revealed partial resolution of the hyperintensity, and complete resolution of the restricted diffusion previously seen in the corpus callosum, which confirmed the diagnosis of mild encephalitis/encephalopathy with a reversible splenial lesion. We highlight the importance of recognizing this phenomenon in association with SARS-CoV-2 infection.

## Introduction

Encephalopathy secondary to severe acute respiratory syndrome coronavirus 2 (SARS-CoV-2) infection has been reported in the literature, with an estimated prevalence ranging from 7 to 69% [1-2]. It is more common in patients who are 50 years of age or older or those who are critically ill [1-2]. Neuroimaging in these patients is typically unremarkable, but some abnormal findings have been reported, including T2/fluid-attenuated inversion recovery (FLAIR) hyperintensities in the cortical and/or subcortical white matter, posterior reversible leukoencephalopathy, microhemorrhages, or leptomeningeal enhancement [1-2]. To date, only a few cases have been described in the literature in which SARS-CoV-2 infection is associated with mild encephalopathy with a reversible splenial lesion (MERS), also known as cytotoxic lesions of the corpus callosum (CLOCCs) [3-5].

MERS has a monophasic course of encephalopathy with marginal pleocytosis in the cerebrospinal fluid (CSF), and a characteristic MRI finding of a reversible lesion in the splenium of the corpus callosum (SCC) [6]. This article reports a case of a patient presenting with altered mental status who tested positive for SARS-CoV-2 infection and was diagnosed with MERS. A literature review of related cases is also included.

## Case presentation

A 68-year-old woman with a history of type 2 diabetes mellitus, hypertension, and hyperlipidemia presented to the emergency department with altered mental status after being found unconscious during a welfare check. Initial neurological evaluation revealed patient was awake, alert, and oriented to name and age but not date and time. Speech was fluent with intact naming, repetition, and comprehension, although poor attention was noted during the encounter. Cranial nerve examination was unremarkable. Motor examination was non-focal, with normal bulk and tone and generalized 4/5 strength throughout. Deep tendon reflexes were decreased bilaterally to 1+ with downgoing plantar reflexes. Sensory and coordination examination was unrevealing. 

Laboratory workup revealed an elevated white blood cell count of 17.4 thou/cmm (4.0-10.0 thou/cmm) and hypernatremia of 147 mmol/L. Urinalysis, blood cultures, respiratory viral panel, streptococcus panel, and urine drug screen were negative. Chest x-ray showed peripheral consolidative opacities in the bilateral upper and lower lobes, and a follow-up CT revealed multiple ground-glass opacities. Polymerase chain reaction (PCR) testing for SARS-CoV-2 was positive. Vaccination history was up-to-date except for SARS-CoV-2.

**Figure 1:**
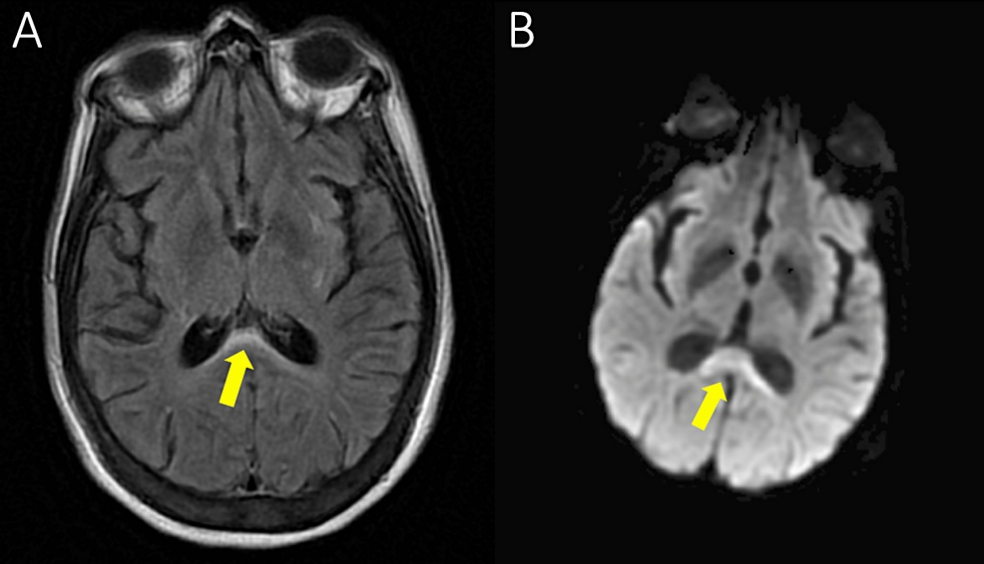
MRI of the brain revealing a hyperintensity on FLAIR (A) with low diffusivity on DWI (B) in the corpus callosum. MRI = Magnetic Resonance Imaging, FLAIR = fluid-attenuated inversion recovery, DWI = diffusion-weighted imaging

CT of the head was unremarkable. MRI of the brain revealed increased signal in the corpus callosum on FLAIR with low diffusivity on diffusion-weighted imaging (DWI) (known as boomerang sign) (Figure 1) with apparent diffusion coefficient (ADC) correlation (images not shown). Furthermore, there was suggestion of loss of normal flow void in the superior sagittal sinus, right transverse sinus, and sigmoid sinuses (images not shown). CT angiogram/venogram, however, showed no evidence of cerebral venous sinus thrombosis.

**Figure 2:**
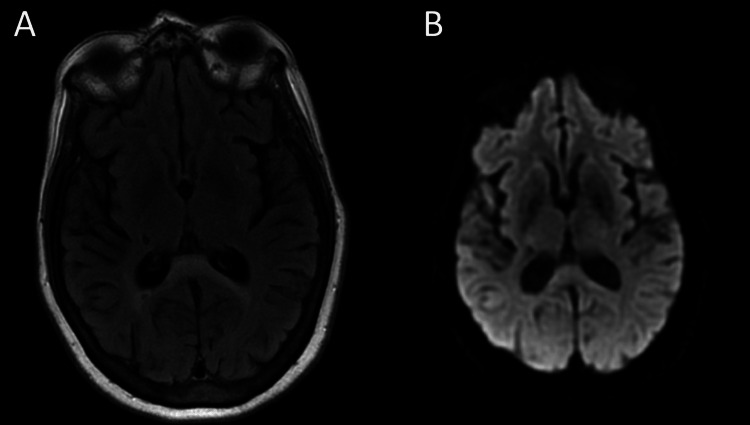
Repeat MRI of the brain revealing partial resolution of the hyperintensity (A) and complete resolution of low diffusivity (B) in the corpus callosum. MRI = Magnetic Resonance Imaging

The patient was treated with tocilizumab, dexamethasone, remdesivir, and ceftriaxone over a period of five days to treat superimposed bacterial pneumonia. Acute kidney injury and hypernatremia improved with intravenous fluids. Toxic-metabolic encephalopathy was the leading differential diagnosis at this point. The patient’s mentation and clinical status improved gradually throughout the following week of hospitalization and she eventually was transitioned to an inpatient rehabilitation facility. The patient significantly improved and repeat brain MRI five weeks from initial imaging showed improvement of the hyperintensity and resolution of the DWI abnormality in the SCC (Figure 2), supporting the diagnosis of MERS.

## Discussion

Over the past couple of years, there has been a broad spectrum of neurological manifestations of SARS-CoV-2 infection including headache, dizziness, anosmia, and altered mental status [7]. More severe and less common neurological manifestations include acute ischemic stroke, intracerebral hemorrhage, central sinus venous thrombosis, and seizures [7]. Encephalopathy is a common symptom in patients with SARS-CoV-2 infection [8-9]. Though more common in critically ill patients, it can also be seen in patients with mild infection. Kennedy et al. reported that 37% of patients who presented with altered level of consciousness lacked typical symptoms associated with SARS-CoV-2 such as fever or dyspnea. Risk factors for encephalopathy include older age, vision impairment, history of Parkinson’s disease or stroke, and prior psychoactive medication use [9]. Several cases of patients in pediatric and adult populations with acute SARS-CoV-2 infection presenting with MERS or CLOCCs have been reported [3-5,8-11]. Three cases of MERS following SARS-CoV-2 vaccination have also been reported [10-11].

MERS has been well-described in children but rarely reported in adults. Apart from encephalopathy, patients with MERS had other neurological complications such as seizures and cranial nerve dysfunction, even with isolated lesion(s) in the splenium [6]. It has been related to numerous infectious agents including the influenza virus, measles, O-157 Escherichia coli, and Mycoplasma pneumonia [12-13].

Hayashi et al. reported the first presumed case of MERS associated with SARS-CoV-2 infection in April 2020 [3]. A 75-year-old man presented with disorientation and was found to have finger-to-nose dysmetria on neurological examination. MRI of the brain showed an abnormal hyperintensity in the SCC on DWI, and patient tested positive for SARS-CoV-2 in the setting of recent exposure. The ataxia resolved on hospital day two and mental status improved by day four. Like our patient, he was treated for SARS-CoV-2 infection and did receive antibiotics. However, repeat brain imaging was not completed because the patient died on day 12 due to respiratory failure [3]. Therefore, it is unsure if the corpus callosum lesion was indeed reversible. The second case was reported in June 2020 of a 69-year-old man who presented with fever and encephalopathy following travel to Nigeria [4]. Neurological examination was notable for disorientation, impaired awareness, and bradyphrenia. Brain MRI revealed a non-enhancing region of restricted diffusion and FLAIR hyperintensity in the SCC. Unlike our case and the previous case, this patient’s PCR testing for SARS-CoV-2 was negative. However, CSF testing did show elevated SARS-CoV-2 IgA and IgG antibodies suggesting a recent exposure. Mental status improved over two weeks and repeat MRI brain imaging demonstrated resolution of the corpus callosum lesion. This was a presumed case of MERS associated with SARS-CoV-2 due to the presence of antibodies [4]. In both cases, neurological deficit was the presenting symptom followed by a positive PCR that led to diagnosis of SARS-CoV-2-associated MERS.

Bektas et al. described two pediatric cases in October 2020 of a 10-year-old boy and an 11-year-old girl who both presented with diarrhea, fever, rash, and agitation [5]. Though they tested negative for SARS-CoV-2 PCR, reverse transcription (RT)-PCR was positive for anti-SARS-CoV-2 IgM and IgG a week later. Neurological symptoms resolved within three to four days and brain MRI on day seven was normal. Unlike our patient, they received intravenous immunoglobulin (IVIG) followed by high-dose steroids with the diagnosis of multisystem inflammatory syndrome [5]. Compared with our patient and the prior two cases reported by Hayashi et al. and Kakadia et al., improvement of neurological deficits and resolution of radiographic abnormalities in these two pediatric cases was more rapid, occurring within seven days. Both patients received immunosuppression including IVIG and corticosteroids, which may alter the pro-inflammatory response that has been proposed to trigger MERS.

A cytokine-driven inflammatory process precipitating cellular swelling and cytotoxic edema has been proposed as the underlying pathophysiology of MERS [10]. Fong et al. reported that serial diffusion tensor imaging demonstrated normal fractional anisotropy values upon resolution of the splenial lesion, suggesting that MERS was most likely the result of transient interstitial edema with relative preservation of white matter tracts [14]. Aiba et al. reported that serum levels of interleukin (IL)-6 significantly correlated with the clinical severity and prognosis of this condition [15]. Morichi et al. identified elevated levels of interleukin-10 and interferon-γ in the CSF during early stages of viral-induced MERS [16]. However, these findings were reported in the pediatric population and not routinely tested in other cases [16-17]. SARS-CoV-2 infection is known to trigger an inflammatory response, with severe cases leading to cytokine storm [18]. Recent studies have reported hyperproduction of mainly pro-inflammatory cytokines, including IL-1, IL-6, IL-12, interferon-γ (IFN-γ), and tumour necrosis factor α (TNFα) [18-19]. This may also play a role in the underlying pathophysiology of developing MERS.

## Conclusions

We present a rare case of MERS triggered by SARS-CoV-2 in a patient who presented with encephalopathy. This case expands the clinical spectrum of neurological manifestations and radiographic abnormalities associated with SARS-CoV-2 and adds to the already existing literature. Toxic-metabolic encephalopathy is a common clinical manifestation in patients with SARS-CoV-2 infection. When patients do not improve within days, additional diagnostic testing should be pursued. However, as seen in our case, encephalopathy associated with MERS triggered by SARS-CoV-2 could take days to weeks to resolve. Furthermore, the prevalence of MERS in patients with SARS-CoV-2 remains undetermined. Further investigation is thereby warranted for a better understanding of the prevalence, pathophysiology, and management of this entity to avoid unnecessary diagnostic and therapeutic interventions.
